# Association between serum vitamin D levels and cardiovascular disease risk in patients with hyperuricemia: A cross-sectional study

**DOI:** 10.1371/journal.pone.0346141

**Published:** 2026-04-03

**Authors:** Huan Lei, Biyue Wei, Xiao Dong, Meiling Jia, Xiaoyu Huang, Jingshu Yan, Furong Li, Meihua Liang

**Affiliations:** Department of Endocrinology and Metabolism, The Second Affiliated Hospital of Harbin Medical University, Heilongjiang, China; Fraunhofer USA, Inc. Center Midwest, UNITED STATES OF AMERICA

## Abstract

Hyperuricemia (HUA) is an independent risk factor for cardiovascular disease (CVD), and low serum vitamin D levels are associated with an increased risk of CVD. However, their combined effect in patients with HUA has not been well characterized. This study aimed to investigate the association between circulating vitamin D levels and CVD in patients with HUA, examine whether vitamin D deficiency (VDD) is associated with increased CVD prevalence, and determine the correlation between low vitamin D levels and CVD severity. The study employed a cross-sectional design and included clinical data of 483 patients with HUA admitted to the Second Affiliated Hospital of Harbin Medical University between 01/09/2023 and 01/11/2024. According to the criteria by the National Academy of Medical Sciences, participants were stratified into three groups based on serum 25-hydroxyvitamin D (25(OH)D) concentrations: VDD group (< 12 ng/mL, n = 150), vitamin D insufficiency group (12 ng/mL ≤ 25(OH)D < 20 ng/mL, n = 226), and vitamin D sufficiency group (≥ 20 ng/mL, n = 107). CVD prevalence was higher in the VDD group than in the vitamin D sufficiency group (*p* < 0.05), and the Gensini score—which reflects the degree of coronary stenosis—was higher in the VDD group than in the vitamin D sufficiency group (*p* < 0.05). Spearman’s rank correlation analysis showed that 25(OH)D was negatively correlated with glycated hemoglobin (r = −0.154, *p* = 0.004), total cholesterol (r = −0.181, *p* < 0.001), triacylglycerol (r = −0.202, *p* < 0.001), and Gensini score (r = −0.27, *p* = 0.002). Logistic regression identified vitamin D (odds ratio [OR] = 0.94, 95% confidence interval [CI] [0.90–0.97], *p* = 0.001) and age (OR = 1.06, 95% CI [1.03–1.09], *p* < 0.01) as influential factors for CVD. Among patients with HUA, those with VDD exhibited a higher CVD prevalence and greater coronary artery stenosis compared to those with sufficient vitamin D levels. Vitamin D status was independently associated with a reduced risk of CVD, while age with an increased risk of CVD, in patients with HUA.

## Introduction

Hyperuricemia (HUA) is a metabolic disorder characterized by elevated serum uric acid levels, resulting from excessive endogenous and exogenous uric acid production coupled with inadequate renal and intestinal excretion [1]. HUA is a globally prevalent disease, with the prevalence ranging from 2.6% to 36% [2]. In China, it ranks second among common metabolic disorders after diabetes [3]. HUA has been identified as an independent risk factor for cardiovascular disease (CVD) [4,5], and serum uric acid levels exhibit a U-shaped relationship with the prevalence and mortality of CVD [6,7]. Elevated urea levels have been well established to correlate with CVD [8–10], potentially through activating the renin-angiotensin system, inhibiting nitric oxide synthesis, and triggering oxidative stress and pro-inflammatory pathways, which consequently cause endothelial dysfunction and accelerate atherosclerosis [11].

CVD is the leading cause of death globally and ranks first in disease-specific mortality among both urban and rural populations in China [12]. Due to CVD’s complex pathogenesis and severe health consequences—which also impose a heavy burden on national healthcare systems—no effective preventive strategies or interventions currently exist to mitigate this potential risk. Therefore, attention to such patients and the identification of relevant predictive factors are important.

Over the past century, the focus on vitamin D deficiency (VDD) has shifted from nutrition to endocrinology, and in the last two decades, it has returned to nutritional research owing to its pleiotropic effects beyond bone health [13]. The global prevalence of VDD ranges from 20% to 90% [12], affecting over one billion people [14]. Elevated prevalence rates are observed even in regions with abundant sunlight. Furthermore, since vitamin D receptors are widely expressed in the circulatory system [15–17], including cardiomyocytes, vascular smooth muscle cells, and endothelial cells [13], vitamin D has been linked to various diseases, including CVD.

Vitamin D testing is economical and rapid, and multiple observational studies and meta-analyses have demonstrated an inverse correlation between serum 25-hydroxyvitamin D (25(OH)D) levels and CVD risk [18–21]. However, existing large-scale randomized controlled trials (RCTs) remain inconclusive regarding whether increased vitamin D levels reduce the incidence of major cardiovascular events, such as myocardial infarction, stroke, and cardiovascular mortality [12,18]. For example, results from the large VITAL (United States) [22–24] and ViDA (New Zealand) [25–27] studies suggested that vitamin D supplementation does not decrease the risk of CVD. Finland’s FIND trial [28,29] also failed to note a reduction in the number of major cardiovascular events. Nevertheless, the latest findings from the ViDA and FIND studies suggested some modest benefits regarding central blood pressure and atrial fibrillation. In the D-Health (Australia) trial [30–32], despite a small absolute risk difference, the overall incidence of major cardiovascular events was lower in the intervention group than in the placebo group.

Notably, existing research on vitamin D and CVD has primarily focused on the general population or patients with diabetes [33–35], with scarce data on patients with HUA. HUA often coexists with metabolic disorders and renal impairment [36–38], which may interact with vitamin D metabolism to modulate CVD risk. HUA can inhibit the expression of 1-α hydroxylase protein and mRNA, thereby reducing the concentration of 1,25(OH)₂D [39], while VDD may exacerbate HUA-induced inflammatory responses and endothelial dysfunction. Whether vitamin D is associated with CVD risk in patients with HUA remains to be investigated.

Therefore, this study aimed to determine the prevalence of VDD in a cohort of patients with HUA and investigate the correlation between circulating vitamin D levels and CVD. Specifically, we sought to examine whether low vitamin D levels are associated with an increased prevalence of CVD in patients with HUA, explore the correlation between low vitamin D levels and CVD severity (assessed by Gensini score [GS], segment involvement score [SIS], and segment stenosis score [SSS]), and identify the influencing factors for CVD in this population. The findings of this study are expected to provide a more effective basis for the clinical diagnosis, risk stratification, and targeted interventions for CVD in patients with HUA.

## Materials and methods

### Study selection

**Fig 1:**
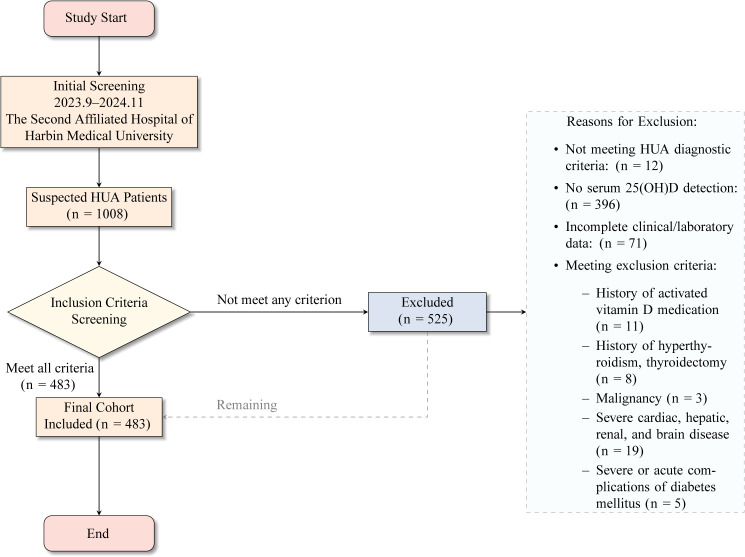
Flowchart of patient selection and grouping.

Data were collected from 483 patients with HUA admitted to the cardiology, neurology, and endocrinology departments of the Second Affiliated Hospital of Harbin Medical University between 01/09/2023 and 01/11/2024. The study period covered four seasons to reduce seasonal bias in vitamin D levels. Patients were categorized into three groups based on the National Academy of Medicine (formerly Institute of Medicine) criteria, consistent with the routine clinical reference range of our hospital: VDD (serum 25(OH)D < 12 ng/mL; n = 150), vitamin D insufficiency (12 ng/mL ≤ 25(OH)D < 20 ng/mL; n = 226), and vitamin D sufficiency (25(OH)D ≥ 20 ng/mL; n = 107) groups. Among the participants, 374 had CVD and 109 did not. The collected data included general characteristics, laboratory test results, and CVD-related examination findings. The study adhered strictly to ethical guidelines, and written informed consent was obtained from all participants. All data were anonymized to ensure participant privacy. The study was approved by the Medical Ethics Committee of the Second Affiliated Hospital of Harbin Medical University (Approval Number: YJSKY2023−370). A flowchart detailing patient screening, inclusion, and exclusion is presented in Fig 1.

### Inclusion criteria

The inclusion criteria were as follows: (1) patients meeting the diagnostic criteria for HUA in the 2023 Chinese Multidisciplinary Expert Consensus on the Diagnosis and Treatment of Diseases Related to HUA, i.e., non-same-day fasting blood uric acid level > 420 μmol/L on a regular purine diet; (2) patients who underwent serum vitamin D testing; (3) patients diagnosed with CVD according to the International Statistical Classification of Diseases and Related Health Problems, 11th edition, diagnostic criteria; the CVDs include coronary artery disease—a combination of history, physical examination, electrocardiogram, laboratory tests, invasive coronary angiography (ICA), and coronary computed tomography angiography (CCTA)—and stroke (Encompasses patients with chronic, stable ischemic/hemorrhagic stroke who were in the convalescent phase and had no acute exacerbation at the time of enrollment)—ischemic or hemorrhagic stroke clinically diagnosed by history, physical examination, laboratory tests, cranial magnetic resonance imaging, cranial computed tomography angiography, and other auxiliary tests; and (4) complete clinical data and test results.

### Exclusion criteria

The exclusion criteria were as follows: (1) use of xanthine oxidase inhibitors, cyclosporine, and other drugs that affect uric acid metabolism in the last 6 months preceding the study; (2) severe metabolic abnormalities of the liver and kidney, severe malignant arrhythmia and heart failure, and severe or acute cranial or cerebral complications (e.g., acute stroke within 2 weeks of enrollment, acute intracranial hemorrhage, severe traumatic brain injury, acute encephalitis); (3) use of medications affecting the metabolic level of vitamin D, such as bisphosphonates, activated vitamin D, calcitonin, estrogens, and estrogen receptor modulators, in the last 6 months preceding the study; (4) history of illnesses or conditions affecting the level of vitamin D—for example, hyperthyroidism, hypothyroidism, hyperparathyroidism, hypoparathyroidism, history of resection, malignant tumors, malignant infectious diseases, other systemic inflammatory diseases, severe or acute complications of diabetes mellitus, Cushing syndrome, osteogenesis imperfecta, osteochondrosis, pregnancy, and breastfeeding; or (5) history of vitamin D or serum uric acid metabolism disorders.

### Data collection

#### General characteristics.

Data on sex, age, height, weight, body mass index (BMI), systolic blood pressure (SBP), diastolic blood pressure (DBP), heart rate (HR), medical history, diabetes history, hypertension history, smoking history, and drinking history of all participants were collected.

#### Laboratory data.

Laboratory data were obtained from venous blood samples collected from study participants at least 8 h after an overnight fast. For the analysis of biochemical parameters including 25-hydroxyvitamin D, creatinine (Cr), serum calcium (Ca), serum phosphorus (P), fasting plasma glucose (FPG), lipids, and lactate dehydrogenase (LDH), 5 mL of venous blood was drawn into serum separator tubes. After clotting at room temperature for 30–60 minutes, the samples were centrifuged at 3000 × g for 10 minutes to isolate serum, and all serum separations were completed within 2 hours of phlebotomy. For the determination of glycated hemoglobin (HbA1c), cardiac troponin I (cTnI), and brain natriuretic peptide (BNP), 3 mL of whole blood was collected into EDTA-K₂ anticoagulant tubes. Plasma was separated for cTnI and BNP measurements, while HbA1c was analyzed directly using fresh whole blood.

The analytical methods, instrumentation, and reference ranges for each parameter were as follows:

Vitamin D (serum 25(OH)D): Detected by chemiluminescence using an Architect i2000 analyzer (Abbott Laboratories, USA) with the Architect 25-OH Vitamin D Reagent Kit (Cat. No.: 5P02; Abbott Laboratories, Abbott Park, IL, USA). The analytical sensitivity (LoD) of the assay was 2.2 ng/mL (5.5 nmol/L), and the lower limit of quantitation (LoQ) was 2.4 ng/mL (6.0 nmol/L). The assay showed high analytical specificity, with cross-reactivity of 98.6% to 101.1% for 25-hydroxyvitamin D3. Reference ranges were defined as: deficiency (< 12 ng/mL), insufficiency (12–20 ng/mL), and sufficiency (≥ 20 ng/mL);

BNP and cTnI: Detected by chemiluminescence using a Dimension analyzer (Siemens Healthineers, Germany). Reference ranges were defined as: cTnI (0–0.056 μg/L), BNP (0–125 pg/mL);

HbA1c: Detected by high-performance liquid chromatography using an ADAMS A1c HA-8180 analyzer (ARKRAY, Inc., Japan). The reference range was 4.0%–6.0%;

Uric acid, Cr, Ca, P, FPG, total cholesterol (TC), triglycerides (TG), low-density lipoprotein cholesterol (LDL-C), high-density lipoprotein cholesterol (HDL-C), and LDH: Analyzed on a cobas c702 automatic biochemical analyzer (Roche Diagnostics, Switzerland). Reference ranges were defined as: uric acid (150–440 μmol/L, detected by colorimetry), Cr (male: 57–97 μmol/L; female: 39–76 μmol/L, detected by enzymatic kinetic method), Ca (2.1–2.8 mmol/L, detected by enzymatic kinetics method), P (0.8–1.4 mmol/L, detected by colorimetry), FPG (3.9–6.1 mmol/L, detected by enzymatic method), TC (1.80–5.17 mmol/L, detected by enzymatic method), TG (0.56–1.70 mmol/L, detected by enzymatic method), HDL-C (1.04–1.70 mmol/L, detected by direct method), LDL-C (0.45–3.15 mmol/L, detected by direct method), and LDH (120–250 U/L, detected by rate method).

All assays were performed strictly in accordance with the manufacturers’ instructions.

#### CVD-related examination data.

All ICA and CCTA findings were collected, including the location and severity of coronary artery lesions (classified as mild, moderate, or severe or quantified by stenosis percentage). The degree of coronary artery stenosis in patients with coronary artery disease was assessed using three scoring systems: GS [40], SIS, and SSS [40]. A higher score indicates more severe coronary artery stenosis.

The GS was applied to all patients who underwent ICA. The scoring algorithm is defined as follows: coronary artery stenosis ≤ 25% corresponds to 1 point; 26–50%, 2 points; 51–75%, 4 points; 76–90%, 8 points; 91–99%, 16 points; and 100%, 32 points. A location-based weighting system was additionally adopted: the left main trunk was assigned 5 points; proximal segment of the left anterior descending artery or circumflex artery, 2.5 points; mid-segment of the left anterior descending artery, 1.5 points; distal segment of the left anterior descending artery, right coronary artery, posterior lateral branch, posterior descending artery, obtuse marginal branch, and first diagonal branch, 1 point; and all other branches, 0.5 points. For each participant, the total score was calculated by multiplying the scores of all diseased vessels and summing the results.

The SIS and SSS were utilized for all patients who underwent CCTA. The SIS divides the coronary artery tree into 16 segments based on the modified American Heart Association criteria: left main artery (LM); proximal, middle, and distal segments of the left anterior descending artery (LAD); proximal and distal segments, first diagonal branch, second diagonal branch, first obtuse marginal branch, second obtuse marginal branch of the left circumflex artery (LCX); proximal, middle, and distal segments of the right coronary artery (RCA); and left posterior branch, right posterior branch, and posterior descending artery. Each affected segment of the coronary arteries is assigned 1 point, with the total score ranging from 0 to 16 points. The SSS assigns weighting coefficients based on the degree of lumen stenosis in each affected segment of the coronary arteries: normal is assigned 0 points; mild, 1 point; moderate, 2 points; and severe, 3 points, with the total score ranging from 0 to 48 points.

In addition, the extent of vascular lesions was evaluated in terms of two parameters: the cumulative number of major coronary artery branches and the plaque outcomes in three vessels. The cumulative number of major coronary artery branches was utilized for all patients who underwent ICA and CCTA, as follows: single-vessel disease: stenosis ≥ 50% in a single main coronary artery (LAD, LCX, RCA), or stenosis ≥ 50% in any of the three main arteries and their major branches simultaneously; two-vessel disease: simultaneous stenosis ≥ 50% in two main coronary arteries (LM lesions are considered two-vessel disease); and three-vessel disease: simultaneous ≥ 50% stenosis in all the three main vessels.

A 3-vessel plaque outcome was utilized for all patients who underwent CCTA. The 3-vessel plaque outcome was treated as a binary variable to indicate the presence or absence of coronary artery plaques in all the three vessels. If plaques were present in all the three vessels (LAD, LCX, and RCA), the 3-vessel plaque outcome was considered positive; otherwise, it was negative [40].

### Statistical analysis

Statistical analysis was performed using R version 4.3.0. Descriptive statistical methods were used to analyze the data. Normally distributed data are presented as mean ± standard deviation, and group comparisons were performed using t-test. Non-normally distributed data are presented as median and interquartile range, and group comparisons were performed using non-parametric rank-sum tests (Mann–Whitney U-test, Kolmogorov–Smirnov test), with multiple comparisons adjusted using the Bonferroni method. Categorical data are expressed as the number of cases and percentages, and intergroup comparisons were performed using the chi-square (χ²) test. Spearman’s rank correlation analysis was used to explore the correlation between 25(OH)D and various indicators. Univariate logistic regression analysis was used to explore the association between various indicators and the outcome variable (CVD). Multivariate logistic regression analysis with the backward elimination method was used to identify independent risk factors for CVD. Stratified multivariate logistic regression analysis was performed to investigate the heterogeneous associations between 25(OH)D levels and CVD in patients with HUA across subgroups stratified by sex and age. The *p-*value < 0.05 was used to indicate statistical significance.

## Results

### Comparison of general characteristics among 25(OH)D level groups

The levels of 25(OH)D among 483 patients (male, n = 376) followed a skewed distribution (median: 14.90 [11.05–19.80] ng/mL). Patients were divided into three groups based on 25(OH)D levels: 150 cases in the VDD group (median: 9.22 [7.70–10.7] ng/mL), 226 cases in the vitamin D insufficiency group (median: 15.55 [13.70–17.98] ng/mL), and 107 cases in the vitamin D sufficiency group (median: 24.50 [21.70–28.40] ng/mL). Patients were also categorized according to the presence (374 cases) or absence (109 cases) of CVD. The median age of patients was 57.50 (47.25–67.00) years in the VDD group, 59.00 (51.00–66.00) years in the vitamin D insufficiency group, and 60.00 (51.00–67.50) years in the vitamin D sufficiency group. Significant differences in drinking history were observed among the three groups (*p* < 0.05). Further pairwise comparisons revealed no significant differences in drinking history between the groups (*p* > 0.05). Also, no significant differences were observed among the three groups in terms of sex, age, hypertension history, diabetes history, smoking history, SBP, DBP, HR, height, weight, and BMI (*p* > 0.05) (Table 1).

**Table 1:** Comparison of general characteristics of patients with HUA among 25(OH)D level groups.

	N	VDD group (N = 150)	Vitamin D insufficiency group (N = 226)	Vitamin D sufficiency group (N = 107)	H/χ²	*p*
Sex						
Male, n (%)	376	107 (71.33%)	180 (79.56%)	89 (83.18%)	5.82	0.054
Female, n (%)	107	43 (28.67%)	46 (20.44%)	18 (16.82%)		
Age (years)		57.50 (47.25–69.00)	59 (51.00–66.00)	60 (51.00–67.50)	2.16	0.339
History of hypertension						
Yes, n (%)	277	89 (59.33%)	127 (56.44%)	61 (57.01%)	0.32	0.852
No, n (%)	205	61 (40.67%)	98 (43.56%)	46 (42.99%)		
History of diabetes						
Yes, n (%)	211	69 (46.00%)	99 (44.00%)	43 (40.19%)	0.87	0.649
No, n (%)	271	81 (54.00%)	126 (56.00%)	64 (59.81%)		
History of smoking						
Yes, n (%)	69	26 (17.33%)	28 (12.44%)	15 (14.02%)	1.76	0.414
No, n (%)	413	124 (82.67%)	197 (87.56%)	92 (85.98%)		
History of alcohol consumption						
Yes, n (%)	54	24 (16.00%)	23 (10.22%)	7 (6.54%)	6.02	**0.049**#
No, n (%)	428	126 (84.00%)	202 (89.78%)	100 (93.46%)		
SBP (mmHg)		142 (129–160)	141 (127–158)	140 (125–150)	2.32	0.314
DBP (mmHg)		89 (78–100)	90 (80–99)	85 (76–96)	4.56	0.102
HR (beats/min)		82 (76–98)	82 (71–91)	80 (74–90)	3.17	0.205
Height (cm)		170.0 (161.0–174.0)	170.0 (165.0–175.0)	170.0 (165.5–174.5)	2.12	0.346
Weight (kg)		75.0 (63.0–85.0)	76.3 (70.0–85.0)	75.0 (70.0–85.0)	3.15	0.207
BMI (kg/m²)		25.89 (23.49–29.01)	26.24 (24.22–29.49)	26.42 (24.22–28.73)	1.06	0.589

25(OH)D, 25-hydroxyvitamin D; BMI, body mass index; DBP, diastolic blood pressure; SBP, systolic blood pressure; VDD, vitamin D deficiency

# When comparing each pair of the three groups separately, no significant differences in the proportions of these categories were observed at the *p* < 0.05 level.

### Comparison of laboratory data among 25(OH)D level groups

A comparison of laboratory data among the three groups revealed significant differences in vitamin D, HbA1c, TC, and TG levels (*p* < 0.05). Further pairwise comparisons revealed significant differences in vitamin D levels among the three groups (*p* < 0.05). HbA1c, TC, and TG levels differed significantly between the vitamin D deficiency and sufficiency groups (all *p* < 0.05). No significant differences were observed in uric acid, Cr, Ca, P, FBG, HDL-C, LDL-C, cTnI, BNP, and LDH (*p* > 0.05) (Table 2).

**Table 2:** Comparison of laboratory data of patients with HUA among 25(OH)D level groups.

	VDD group (N = 150)	Vitamin D insufficiency group (N = 226)	Vitamin D sufficiency group (N = 107)	H	*p*
Vitamin D (ng/mL)	9.22 (7.70–10.70)^a, b^	15.55 (13.70–17.98) ^c^	24.50 (21.70–28.40)	412.97	< 0.001*
UA (μmol/L)	469.85 (446.25–525.97)	470.25 (439.40–513.77)	472.60 (433.90–526.75)	1.35	0.509
Cr (μmol/L)	84.50 (73.00–105.50)	88.00 (75.25–102.00)	90.00 (78.00–107.00)	2.40	0.301
Ca (mmol/L)	2.36 (2.29–2.42)	2.36 (2.28–2.44)	2.36 (2.29–2.45)	0.89	0.642
P (mmol/L)	1.09 (0.94–1.26)	1.04 (0.88–1.20)	1.06 (0.93–1.19)	4.33	0.115
HbA1c (%)	7.40 (6.00–8.90) ^b^	6.50 (5.90–7.90)	6.40 (5.70–7.90)	9.27	0.010*
FBG (mmol/L)	5.96 (5.09–8.33)	6.00 (5.08–7.33)	5.74 (4.96–7.42)	1.52	0.468
TC (mmol/L)	4.81 (3.87–5.69) ^b^	4.55 (3.73–5.27)	4.03 (3.35–5.18)	12.78	0.002*
TG (mmol/L)	2.17 (1.50–4.10) ^b^	1.85 (1.30–2.98)	1.62 (1.12–2.49)	14.63	< 0.001*
HDL-C (mmol/L)	0.98 (0.83–1.18)	0.99 (0.86–1.15)	0.99 (0.87–1.17)	0.58	0.748
LDL-C (mmol/L)	2.59 (2.08–3.52)	2.76 (2.05–3.34)	2.40 (1.79–3.26)	4.92	0.086
cTnI (μg/L)	0.02 (0.02–0.02)	0.02 (0.02–0.02)	0.02 (0.02–0.02)	2.09	0.351
BNP (pg/mL)	163.00 (43.50–828.75)	76.00 (24.00–354.50)	100.50 (26.75–532.50)	5.12	0.077
LDH (U/L)	194.50 (167.00–235.75)	187.50 (165.00–229.00)	187.00 (163.00–213.00)	2.60	0.273

25(OH)D, 25-hydroxyvitamin D; BNP, brain natriuretic peptide; Ca, calcium; Cr, creatinine; CTnI, cardiac troponin I; FBG, fasting blood glucose; HbA1c, glycated hemoglobin; HDL-C, high-density lipoprotein cholesterol; HUA, hyperuricemia; LDH, lactate dehydrogenase; LDL-C, low-density lipoprotein cholesterol; P, phosphorus; TC, total cholesterol; TG, triglyceride; UA, uric acid; VDD, vitamin D deficiency

^a^comparison between the VDD and vitamin insufficiency.

^b^comparison between the VDD and vitamin D sufficiency groups.

^c^comparison between the vitamin D insufficiency and sufficiency groups.

*After adjusting the significant values using the Bonferroni correction method for pairwise comparisons, the *p* value for vitamin D levels among the three groups was < 0.01, *p* value for HbA1c levels between the VDD and vitamin D sufficiency groups was 0.016, *p* value for TC levels between the VDD and vitamin D sufficiency groups was 0.001, and *p* value for TG levels between the VDD and vitamin D sufficiency group was < 0.01.

### Comparison of CVD-related examination data among 25(OH)D level groups

**Fig 2:**
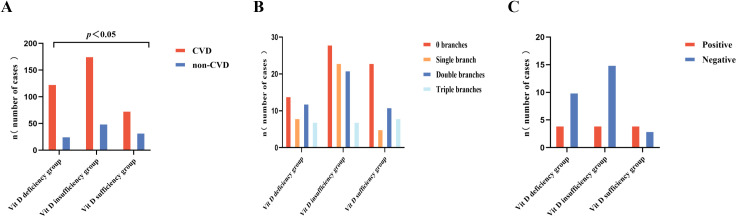
Comparison of CVD-related examination data among 25(OH)D level groups. (A) Comparison of the number of cases with and without CVD among various 25(OH)D level groups; (B) Comparison of cumulative coronary artery branch counts among various 25(OH)D level groups; (C) Number of cases with different 25(OH)D levels and plaque outcomes. 25(OH)D, 25-hydroxyvitamin D; CVD, cardiovascular disease.

**Fig 3:**
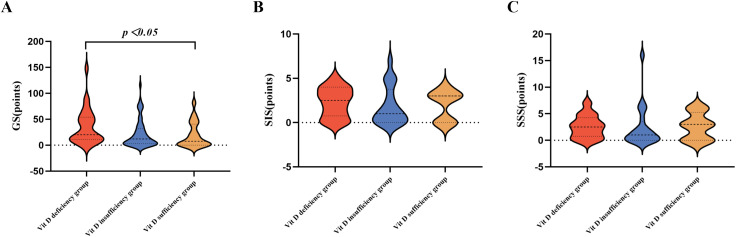
Comparison of CVD-related scores among 25(OH)D level groups. (A) Comparison of GS scores among 25(OH)D level groups; (B) Comparison of SIS scores among 25(OH)D level groups; (C) Comparison of SSS scores among 25(OH)D level groups. 25(OH)D, 25-hydroxyvitamin D; CVD, cardiovascular disease; GS, Gensini score; SIS, segment involvement score; SSS, segment stenosis score.

Significant differences were observed among the three groups in terms of CVD prevalence, proportion of non-CVD individuals, and GS scores (*p* < 0.05). Further pairwise comparisons revealed that CVD prevalence and GS scores were significantly higher in the VDD group than in the vitamin D sufficiency group (*p* < 0.05), and the proportion of non-CVD individuals was significantly lower in the VDD group than in the vitamin D sufficiency group (*p* < 0.05). No significant differences were observed in SIS, SSS, cumulative number of major coronary arteries, and three-vessel plaque outcomes (*p* > 0.05) (Table 3, Fig 2, and Fig 3).

**Table 3:** Comparison of CVD examination data for patients with HUA among 25(OH)D level groups.

	N	VDD group (N = 150)	Vitamin D insufficiency group (N = 226)	Vitamin D sufficiency group (N = 107)	H/χ²	*p*
Outcome indicators						
CVD, n (%)	374	124 (82.76%)^b^	176 (77.88%)	74 (69.16%)	6.57	**0.037** ^#^
non-CVD, n (%)	109	26 (17.33%)^b^	50 (22.12%)	33 (30.84%)		
GS (points)		20.0 (11.0–51.0)^b^	12.0 (3.0–28.0)	7.5 (0.0–40.5)	7.80	0.020*
SIS (points)		2.50 (1.00–3.75)	1.00 (0.00–3.50)	3.00 (1.50–3.00)	0.50	0.780
SSS (points)		2.50 (1.00–3.75)	1.00 (0.00–3.00)	1.00 (0.00–4.50)	1.24	0.539
Number of branches						
0 branches, n (%)	65	14 (34.15%)	28 (35.44%)	23 (48.94%)	8.87	0.181
Single branch, n (%)	36	8 (19.51%)	23 (29.11%)	5 (10.64%)		
Double branches, n (%)	44	12 (29.27%)	21 (26.58%)	11 (23.40%)		
Triple branches, n (%)	22	7 (17.07%)	7 (8.86%)	8 (17.02%)		
3-vessel plaque						
Positive, n (%)	12	4 (28.57%)	4 (21.05%)	4 (57.14%)	–	0.224
Negative, n (%)	28	10 (71.43%)	15 (78.95%)	3 (42.86%)		

25(OH)D, 25-hydroxyvitamin D; CVD, cardiovascular disease; GS, Gensini score; HUA, hyperuricemia; SIS, segment involvement score; SSS, segment stenosis score; VDD, Vitamin D deficiency

^b^Comparison between the VDD and Vitamin D sufficiency groups.

^#^Pairwise comparisons among the three groups at the 0.05 level showed significant differences in column proportions between the VDD and Vitamin D sufficiency groups.

*Pairwise comparisons among the three groups after Bonferroni correction showed significant differences in GS levels between the Vitamin D deficiency and sufficiency groups (*p* value, 0.016).

- Fisher’s exact probability test.

### Correlation analysis between 25(OH)D and various indicators

**Fig 4:**
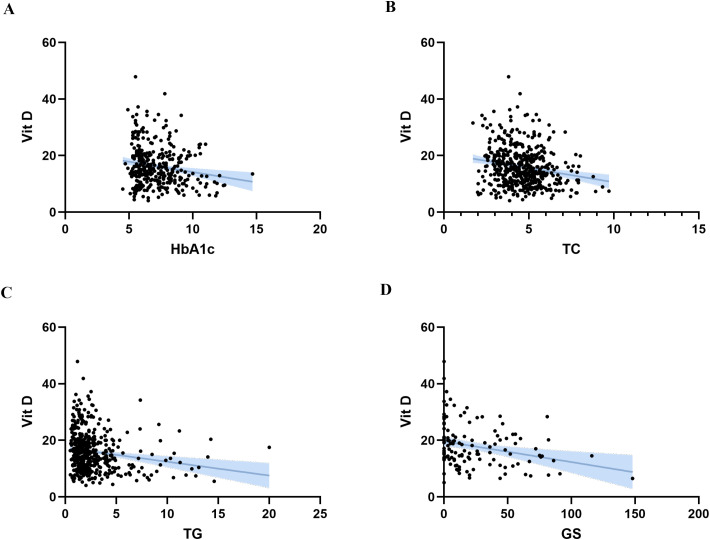
Scatter plot of the correlation analysis between 25(OH)D and various indicators. (A) Scatter plot of HbA1c and 25(OH)D levels; (B) Scatter plot of TC and 25(OH)D levels; (C) Scatter plot of TG and 25(OH)D levels; (D) Scatter plot of GS and 25(OH)D levels. 25(OH)D, 25-hydroxyvitamin D; GS, Gensini score; HbA1c, glycated hemoglobin; TC, total cholesterol; TG, triglycerides.

Spearman’s rank correlation analysis showed that 25(OH)D was negatively correlated with HbA1c (r = −0.154, *p* = 0.004), TC (r = −0.181, *p* < 0.001), TG (r = −0.202, *p* < 0.001), and GS (r = −0.27, *p* = 0.002) (Table 4 and Fig 4).

**Table 4:** Correlation analysis between 25(OH)D and various indicators.

Indicator	r	*p*
HbA1c	−.154	**0.004**
TC	−.181	**< 0.001**
TG	−.202	**< 0.001**
GS	−0.27	**0.002**

25(OH)D, 25-hydroxyvitamin D; GS, Gensini score; HbA1c, glycated hemoglobin; TC, total cholesterol; TG, triglycerides

### Single-factor logistic regression

Univariate logistic regression showed that vitamin D, Cr, P, TC, age, SBP, HR, height, and weight were significantly associated with CVD (*p* < 0.05) (Table 5).

**Table 5:** Single-factor logistic regression results.

	B	SE	Waldχ²	*p*	OR (95%CI)
Vitamin D	−0.04	0.02	−2.83	**0.005**	0.96 (0.93–0.99)
Cr	0.02	0.01	3.21	**0.001**	1.02 (1.01–1.03)
P	−1.25	0.43	−2.90	**0.004**	0.29 (0.12–0.67)
TC	−0.19	0.08	−2.33	**0.020**	0.83 (0.70–0.97)
Age	0.08	0.01	7.62	**< 0.001**	1.08 (1.06–1.10)
SBP	0.01	0.00	1.99	**0.046**	1.01 (1.01–1.02)
HR	−0.02	0.01	−2.10	**0.036**	0.98 (0.97–0.99)
Height	−0.04	0.02	−2.03	**0.042**	0.97 (0.93–0.99)
Weight	−0.03	0.01	−4.25	**< 0.001**	0.97 (0.95–0.98)

CI, confidence interval; Cr, creatinine; HR, heart rate; OR, odds ratio; P, phosphorus; SE, standard error; TC, total cholesterol.

### Multivariate logistic regression

#### Stepwise-adjusted logistic regression models.

To verify the robustness of the association between 25(OH)D and CVD risk, a series of stepwise-adjusted logistic regression models were constructed with incremental inclusion of potential confounding factors. Model 1 (crude model) showed a significant inverse association between 25(OH)D and CVD (OR = 0.96, 95% CI [0.93–0.99], *p* = 0.005). The association remained significant in Model 2, which adjusted for age and sex (OR = 0.95, 95% CI [0.92–0.98], *p* = 0.002). Model 3 incorporated smoking and alcohol consumption, with no substantial change in the association strength (OR = 0.94, 95% CI [0.91–0.97], *p* < 0.001). Model 4 (adjusted for all potential confounding factors) confirmed the robust inverse relationship between 25(OH)D and CVD risk (OR = 0.93, 95% CI [0.90–0.96], *p* < 0.001) (Table 6).

**Table 6:** Stepwise-adjusted logistic regression models.

Variables	Model 1		Model 2		Model 3		Model 4	
OR (95%CI)	*p*	OR (95%CI)	*p*	OR (95%CI)	*p*	OR (95%CI)	*p*
Vitamin D	0.96 (0.93–0.99)	**0.005**	0.93 (0.90–0.96)	**<.001**	0.93 (0.90–0.96)	**<.001**	0.93 (0.90–0.97)	**<.001**

BMI, body mass index; CI, confidence interval; Cr, creatinine; DBP, diastolic blood pressure; HR, heart rate; OR, odds ratio; P, phosphorus; SBP, systolic blood pressure; TC, total cholesterol

Model 1: Crude

Model 2: Adjusted for age and sex

Model 3: Adjusted for age, sex, history of smoking, and history of alcohol consumption,

Model 4: Adjusted for Cr, P, TC, age, sex, history of hypertension, SBP, DBP, HR, diabetes, history of smoking, history of alcohol consumption, height, weight, and BMI

#### Final multivariate logistic regression model.

Variables with *p* < 0.05 in the univariate regression analysis were selected for multivariate regression analysis, and potential confounding factors, such as age, sex, DBP, BMI, HR, history of smoking, history of alcohol consumption, hypertension, and diabetes, were included. The results showed that both vitamin D (OR = 0.94, 95% CI [0.90–0.97], *p* = 0.001) and age (OR = 1.06, 95% CI [1.03–1.09], *p* < 0.01) were significantly associated with CVD (*p* < 0.05). Each 1-unit increase in vitamin D was associated with an approximately 6% reduction in CVD risk, and each additional year of age was associated with an approximately 6% increase in risk (Table 7).

**Table 7:** Multivariate logistic regression results.

	B	SE	Waldχ²	*p*	OR (95%CI)
Vitamin D	−0.07	0.02	10.60	**0.001**	0.94 (0.90–0.97)
Age	0.06	0.01	18.87	**<.001**	1.06 (1.03–1.09)

CI, confidence interval; OR, odds ratio; SE, standard error.

### Stratified analysis by sex and age

To explore the heterogeneity of the association between 25(OH)D levels and CVD in patients with HUA across different sex and age subgroups, we performed stratified multivariate logistic regression analyses. Covariates in each subgroup were consistent with those in the overall population to ensure result comparability. Age was stratified according to the World Health Organization definition of older adults (< 60 vs ≥ 60 years) [41], consistent with the median age of the vitamin D sufficiency group in the cohort, and sex was dichotomized as male or female. Stratified analyses revealed that 25(OH)D level was inversely linked to CVD in both male (OR = 0.95, 95% CI [0.90–0.99], *p* = 0.039) and female (OR = 0.81, 95% CI [0.71–0.93], *p* = 0.003) patients with HUA. Regarding age, the association was significant in the ≥ 60 years group (OR = 0.91, 95% CI [0.85–0.96], *p* = 0.002), but not in the < 60 years group (OR = 0.96, 95% CI [0.90–1.02], *p* = 0.145) (Table 8).

**Table 8:** Sex and age-stratified regression results for 25(OH)D and CVD.

Stratifying factors	Subgroup	n (HUA/ N)	OR (95% CI)	*p*
Sex	Male	376/483	0.95 (0.90–0.99)	**0.039**
	Female	107/483	0.81 (0.71–0.93)	**0.003**
Age (years)	< 60	249/483	0.96 (0.90–1.02)	0.145
	≥ 60	234/483	0.91 (0.85–0.96)	**0.002**

25(OH)D, 25-hydroxyvitamin D; CI, confidence interval; CVD, cardiovascular disease; HU, hyperuricemia; OR, odds ratio.

## Discussion

This study is the first to explore the association between serum 25(OH)D levels and CVD in patients with HUA. Our results not only confirm the significant association between VDD and increased prevalence of CVD and more severe coronary artery disease in patients with HUA, but also lay the foundation for revealing the complex pathophysiological interactions among vitamin D, HUA, and CVD, thereby providing new insights into risk stratification and targeted management strategies in this high-risk population.

### Mechanisms linking Vitamin D, HUA, and CVD

#### Vitamin D and cardiovascular protection.

In addition to its classic role in Ca and P metabolism and bone health, vitamin D also plays a multifaceted cardiovascular protective role [4,42,43]. Numerous epidemiological and basic studies have shown that VDD is associated with an increased risk of various CVDs, such as hypertension, atherosclerosis, heart failure, myocardial infarction, and stroke, through various mechanisms, as follows:

Renin-angiotensin-aldosterone system (RAAS) regulation: Vitamin D, as a negative regulator of RAAS, inhibits renin gene expression and enhances angiotensin-converting enzyme II activity. Consequently, angiotensin II production is reduced [44], thus decreasing vascular tone and arterial stiffness [45]. HUA may activate RAAS and impair endothelial function [46], and VDD may further exacerbate this vicious cycle.

Endothelial function and inflammation: Vitamin D inhibits pro-inflammatory cytokines (e.g., tumor necrosis factor alpha [TNF]-α, interleukin [IL]-6, IL-8, IL-1β, IL-17, and monocyte chemoattractant protein-1) and promotes the release of anti-inflammatory factors (e.g., IL-4 and IL-10) [47] while enhancing endothelial nitric oxide synthase activity and antioxidant enzyme (superoxide dismutase, glutathione peroxidase, and catalase) function [47]. It also stabilizes endothelial cells and inhibits platelet aggregation. However, HUA is characterized by a chronic low-grade inflammatory state that induces NLRP3-dependent inflammatory activation through the AMPK-mTOR-mROS and HIF-1α pathways, leading to a cascade release of inflammatory factors, such as IL-1β [46]. VDD may amplify the inherent inflammatory and endothelial damage effects of HUA, forming a “double whammy” and significantly accelerating atherosclerosis.

Metabolic regulation: Vitamin D regulates inflammation and indirectly protects the cardiovascular system by binding to vitamin D receptors in islet β cells and parathyroid cells, improving insulin sensitivity, inhibiting parathyroid hormone synthesis and secretion, and reducing the risk of insulin resistance and metabolic syndrome [48]. This study revealed significant differences in HbA1c, TC, and TG levels between the VDD and vitamin D sufficiency groups in patients with HUA. In addition, as vitamin D levels decreased, HbA1c, TC, and TG levels gradually increased, consistent with the results of the 2007 NHANES study, which showed an inverse association between vitamin D levels and diabetes, hypertension, hypertriglyceridemia, and obesity. Vitamin D supplementation may reduce serum TC levels by increasing lipoprotein lipase activity in adipose tissues and reduce LDL-C levels by improving calcium absorption and forming insoluble calcium-fatty acid complexes in the gut to minimize fatty acid absorption [45].

Myocardial and vascular integrity: Vitamin D receptors are widely distributed in cardiomyocytes and vascular smooth muscle cells [49]. A decrease in vitamin D levels leads to excessive activation of the RAAS, vascular smooth muscle hyperplasia and fibrosis, and thickening of the myocardium and arteries, which precipitate the progression of CVDs, such as atherosclerosis, endothelial dysfunction, hypertension, and coronary heart disease [44,50,51]. Research on the direct effects of HUA on CVD is limited, but the accompanying inflammation, oxidative stress, and endothelial dysfunction undoubtedly damage cardiomyocytes [46]. VDD may exacerbate the myocardial injury caused by HUA.

#### Bidirectional interaction between Vitamin D and HUA.

Vitamin D and HUA exhibit mutual influences [52], thereby exacerbating CVD risk. VDD induces secondary hyperparathyroidism, reduces renal uric acid clearance through insulin resistance [53], and modulates genes associated with uric acid (SLC2A9, SLC17A3) [54], leading to HUA.

HUA affects vitamin D levels by inhibiting renal 1-α hydroxylase protein and mRNA expression by reducing the concentration of 1,25(OH)2D [53]. It also induces inflammatory cytokines, such as IL-1, IL-6, and TNF-α, to disrupt bone metabolism and is positively correlated with insulin resistance and obesity, leading to obesity-related vitamin D dilution [55].

In addition, HUA-induced VDD may lead to increased parathyroid hormone concentrations, which downregulate the expression of the urate export protein ABCG2 in the gut and kidney, inhibiting uric acid excretion, which in turn leads to urinary flora [53]. In patients with HUA, this bidirectional relationship forms a “vicious circle”: HUA activates RAAS and induces chronic low-grade inflammation [52], while VDD amplifies these effects—together accelerating endothelial damage and atherosclerosis.

### Clinical significance of VDD in patients with HUA

#### Vitamin D as a risk marker for CVD.

This study explored the association of 25(OH)D with CVD for the first time in patients with HUA, and the results showed that VDD was associated with increased CVD prevalence and higher GS scores. This finding is consistent with the results of a meta-analysis of observational studies by Kendrick [19], Gholami [56], Melamed [21], Pencina [57], and Edward [58], which revealed an inverse relationship between serum 25(OH)D levels and CVD risk. Spearman correlation analysis indicated that 25(OH)D was negatively correlated with HbA1c, TC, TG, and GS. Multivariate logistic regression analysis identified vitamin D and age as influential factors for CVD. Each 1-unit increase in vitamin D was associated with an approximately 6% reduction in CVD risk, and each additional year of age was associated with an approximately 6% increase in risk.

These findings are consistent with a report from the NHANES III Chain Death Archive (1988–994), which showed that low 25(OH)D levels (< 17.8 ng/ml) were independently linked to a 26% elevation of all-cause mortality compared with the highest quartile of 25(OH)D among 13,331 adults aged > 19 years [21]. In the report of pilz [59], vitamin D supplementation was associated with a significant 7% decrease in total mortality (summary relative risk 0.93, 95% CI [0.87–0.99]). Among 6,853 patients with CKD, a meta-analysis demonstrated that a 25 nm increase in 25(OH)D levels corresponded to a 14% decreased mortality risk [relative risk 0.86 (95% CI 0.82–0.91)] [60]. Data from the Uppsala Longitudinal Study of Adult Men, involving 1,194 older men, revealed that low/high serum 25(OH)D levels were associated with higher overall and cancer mortality, but only low levels correlated with cardiovascular mortality [61]. A prospective nested case-control study comprising 18,225 men in the United States showed that low vitamin D was associated with a higher risk of myocardial infarction compared with a multivariate-adjusted adequate 25(OH)D [58]. Additionally, in the PoCosteo Study from southern Iran, higher vitamin D concentrations were associated with a 2% decreased risk of dyslipidemia [ 62–64]. These reports support vitamin D as a composite indicator of cardiovascular risk in patients with HUA [13,65]. Meanwhile, advanced age correlates with elevated CVD risk, primarily driven by vascular endothelial dysfunction induced by reduced nitric oxide synthesis and age-related oxidative stress [66].

The sex stratification in this study revealed a significant difference in the protective effect of 25(OH)D on male and female patients with HUA, and the negative correlation between the two was stronger in women. This sex difference is consistent with previous reports showing that vitamin D interacts with 17 β-estradiol to upregulate each other’s receptors [67], the number of vitamin D regulatory genes in women is 3.2 times that in men, and postmenopausal women have a higher rate of VDD [68]. Verdoia et al. reported that the negative cardiovascular effects of VDD may be more relevant in women, and that VDD is associated with increased severity of coronary artery disease in women, but not in men [69]. In postmenopausal female patients with HUA, the synergistic effect of estrogen deficiency and elevated blood uric acid can further impair vascular health.

The age-stratified analysis showed that the negative association between 25(OH)D and CVD was significant in only the patients aged ≥ 60 years. This age-dependent effect is consistent with a previous report showing that older adults face heightened VDD risk due to inadequate dietary intake and impaired cutaneous synthesis [70]. Seals et al. demonstrated that such deficiency modulates age-related vascular endothelial function, thereby elevating the incidence of hypertension [66]. In patients with HUA, age-related renal decline may exacerbate uric acid accumulation and VDD, thereby strengthening the association between 25(OH)D and CVD.

Notably, the differences in SIS, SSS, cumulative number of major coronary branches, and 3-vessel plaque outcome among the three groups were not statistically significant. This finding may reflect the limited sample size of computed tomography angiography-based plaque analysis or suggest that vitamin D primarily affects the overall severity of coronary artery stenosis rather than the number of vessels involved, consistent with the finding of Tardif et al. [62] that VDD is associated with not only the risk of coronary stenosis but also an increased degree of such stenosis [71]. This hypothesis should be further explored through expanded sample sizes, multicenter collaborations, and molecular mechanistic studies.

#### Vitamin D as a surrogate indicator of disease severity.

Low vitamin D levels in patients with HUA suggest increased susceptibility to CVD, as they reflect cumulative exposure to cardiovascular risk factors (inadequate sunlight, obesity, and inflammation) [72], enhanced pathogenic pathway activity (renin-angiotensin system activation and endothelial dysfunction) [73], and more severe metabolic disorders (insulin resistance) [74,75]. The negative correlation between 25(OH)D and GS highlights the utility of vitamin D in identifying patients with HUA with severe coronary stenosis, aiding in risk stratification and targeted management (e.g., uric acid reduction, cardiovascular risk factor control, and potential vitamin D supplementation).

#### Potential applications.

This study lays the groundwork for translational research on vitamin D supplementation in patients with HUA, with one of the key priorities being refinement of the target population. HUA subgroups most likely to benefit from supplementation (e.g., specific uric acid ranges, comorbidities, baseline vitamin D levels) should be identified. From a clinical perspective, our data suggest that vitamin D status may be a useful risk stratification component for patients with HUA. Lower serum 25(OH) D levels can help identify subgroups of HUA with a significantly higher prevalence of CVD and more severe coronary artery disease, necessitating closer monitoring and active management of traditional cardiovascular risk factors.

In addition, rigorous trials are needed to evaluate whether correcting VDD improves endothelial function, decreases inflammatory markers, and reduces major adverse cardiovascular events in patients with HUA while monitoring safety (e.g., risk of HUA in patients with renal insufficiency).

### Limitations

First, the cross-sectional design of the study limits our ability to infer a causal relationship between low vitamin D levels and CVD. Second, this study was retrospective and did not include vitamin D supplementation to further explore whether it can reduce the prevalence of CVD in patients with HUA. Third, although we adjusted for some confounding factors, other unmeasured confounders, such as sun exposure, seasonal variations, and physical activity levels, may still affect the results. Fourth, this study was performed in a single center with a potentially small sample size, which may limit the generalizability of the results.

## Conclusion

This study revealed for the first time that serum 25(OH)D levels in patients with HUA were significantly negatively correlated with the prevalence of CVD, and VDD was associated with a higher prevalence of CVD and more severe coronary artery stenosis. Sex- and age-stratified analyses further showed that 25(OH)D was more protective against CVD in women and patients aged ≥ 60 years. Vitamin D status and age were key factors in the development of CVD in patients with HUA, suggesting that low vitamin D levels may be independently associated with an increased risk of prevalent CVD in this population and can serve as an effective biomarker for identifying high-risk individuals. Future prospective research should focus on accurately quantifying the predictive value of vitamin D for CVD risk in patients with different severities of HUA. Mechanistic studies should also be undertaken to elucidate the effect of VDD on vascular, cardiac, and metabolic homeostasis in patients with HUA. Further, high-quality RCTs should be conducted to determine the optimal target level of vitamin D supplementation and individualized dosage regimens and validate its clinical efficacy in preventing or delaying the onset and progression of CVD in patients with HUA.
